# Supplementary material for: Cerebellum-enriched protein INPP5A contributes to selective neuropathology in mouse model of spinocerebellar ataxias type 17
Source: Nat Commun. 2020 Feb 27;11:1101. doi: 10.1038/s41467-020-14931-8 (PMC7046734; doi:10.1038/s41467-020-14931-8)
Supplement: Supplementary file 1 — Supplementary information. [file 41467_2020_14931_MOESM1_ESM.pdf]

Supplementary information

Cerebellum-enriched protein INPP5A contributes to selective neuropathology in mouse model of Spinocerebellar ataxias type 17

Liu et al.

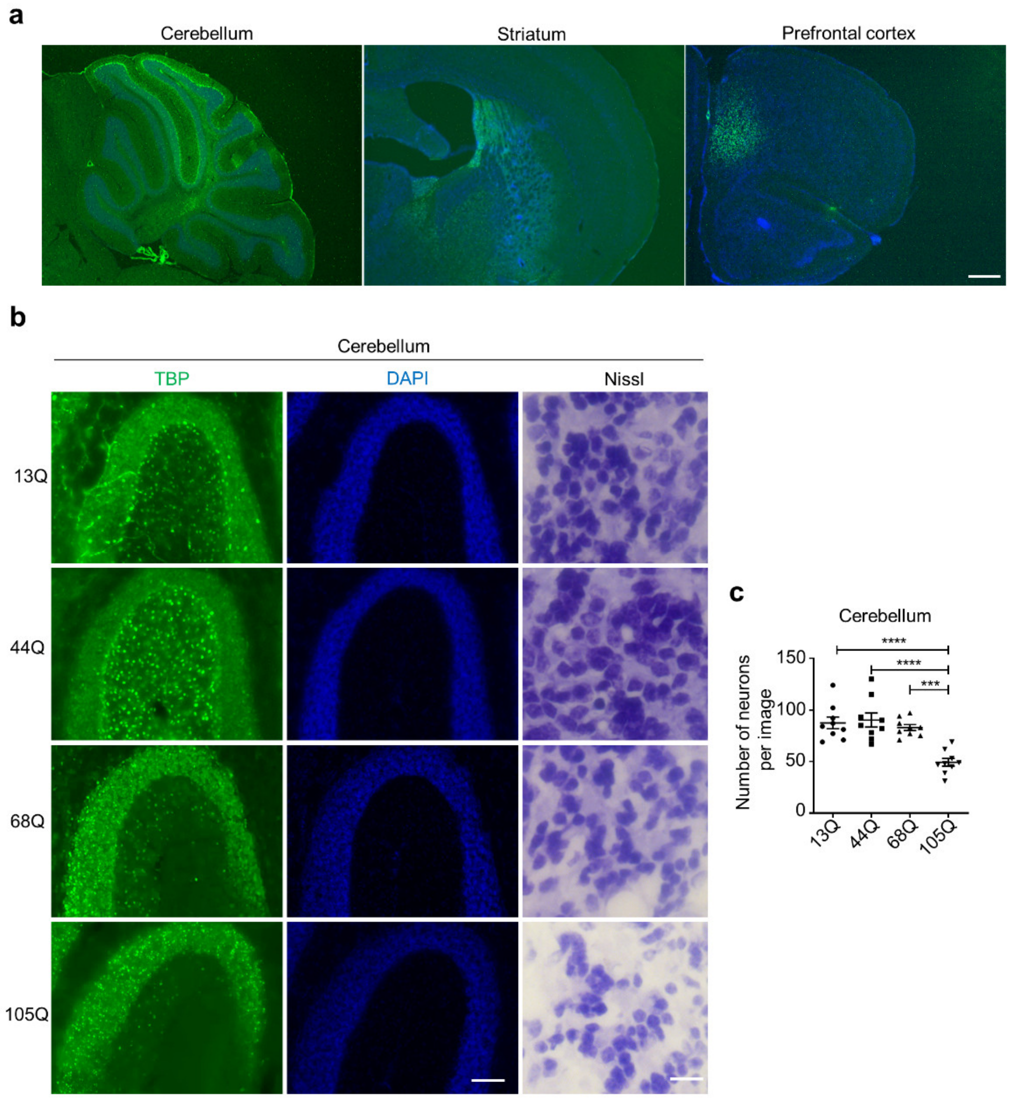

Supplementary Figure 1 Overexpression of mutant *TBP* in different brain regions. **a** Representative immunofluorescence images showing the transduction of AAV-*TBP* in the cerebellum, striatum and prefrontal cortex. Scale bar = 20 μm. **b** Immunofluorescence staining (Scale bar = 100 μm) and Nissl staining (Scale bar = 10 μm) indicated that TBP-105Q dramatically decreased the number of neuronal cells in the cerebellum. **c** The number of Nissl-stained cells per image is presented as mean ± SEM. One-way ANNOVA followed with Tukey's multiple comparisons test was performed, F = 14.36, *** $P < 0.0005$, **** $P < 0.0001$. Data are represented as mean ± SEM. n = 3 mice per group, three images were used to count from each mouse. Source data are provided as a Source Data file.

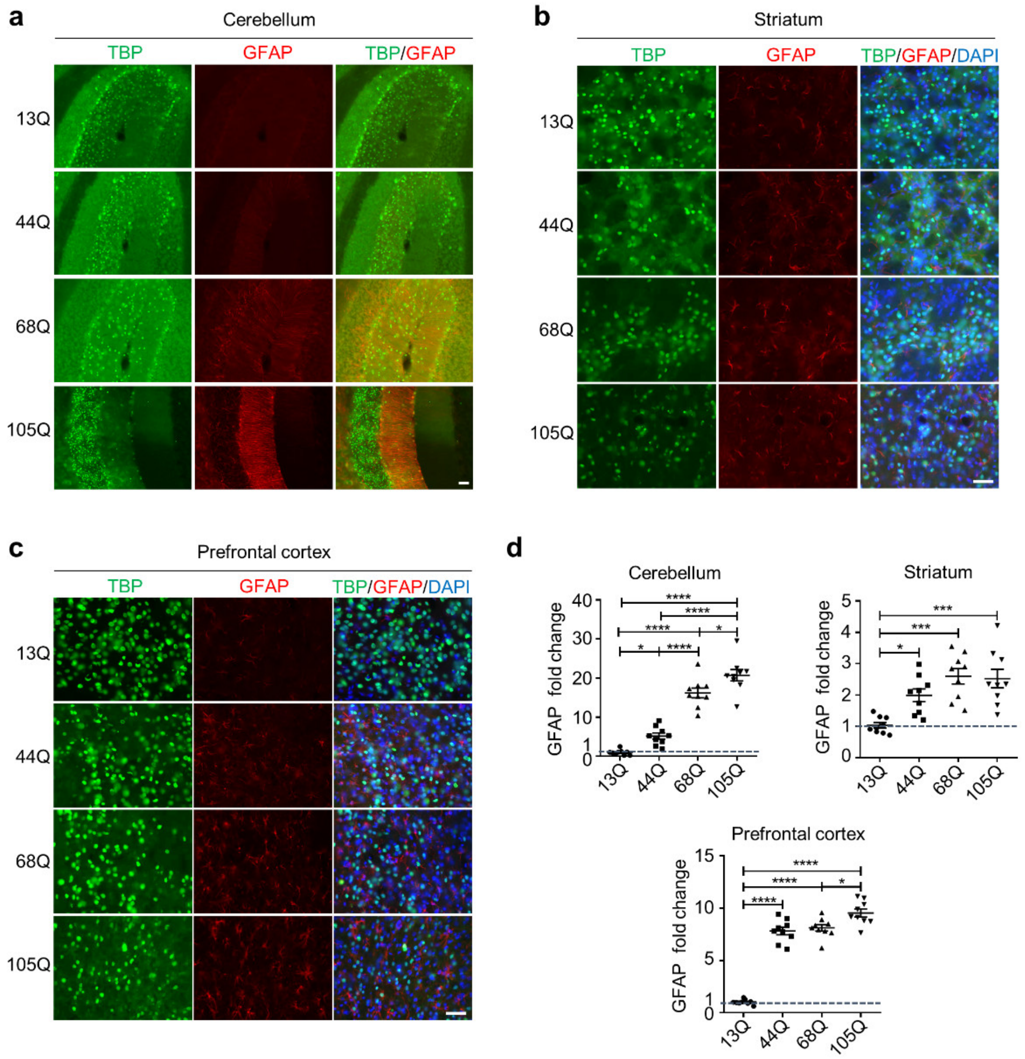

Supplementary Figure 2 Overexpression of mutant *TBP* causes increased reactive astrogliosis in different brain regions. **a-c** Immunofluorescence staining showing increased reactive astrogliosis in a polyQ length dependent manner in the cerebellum (**a**), striatum (**b**) and prefrontal cortex (**c**). Scale bar = 50 µm. **d** Quantification of GFAP staining intensity. Data are presented as fold change compared to TBP-13Q (dashed line) in the cerebellum, striatum, and prefrontal cortex (mean ± SEM). One-way ANNOVA followed with Tukey's multiple comparisons test was performed, cerebellum, F = 78.13; striatum, F = 10.58; prefrontal cortex, F = 152; * $P < 0.05$, *** $P < 0.0005$, **** $P < 0.0001$. Data are represented as mean ± SEM. n = 3 mice per group, three images were used to count from each mouse. Source data are provided as a Source Data file.

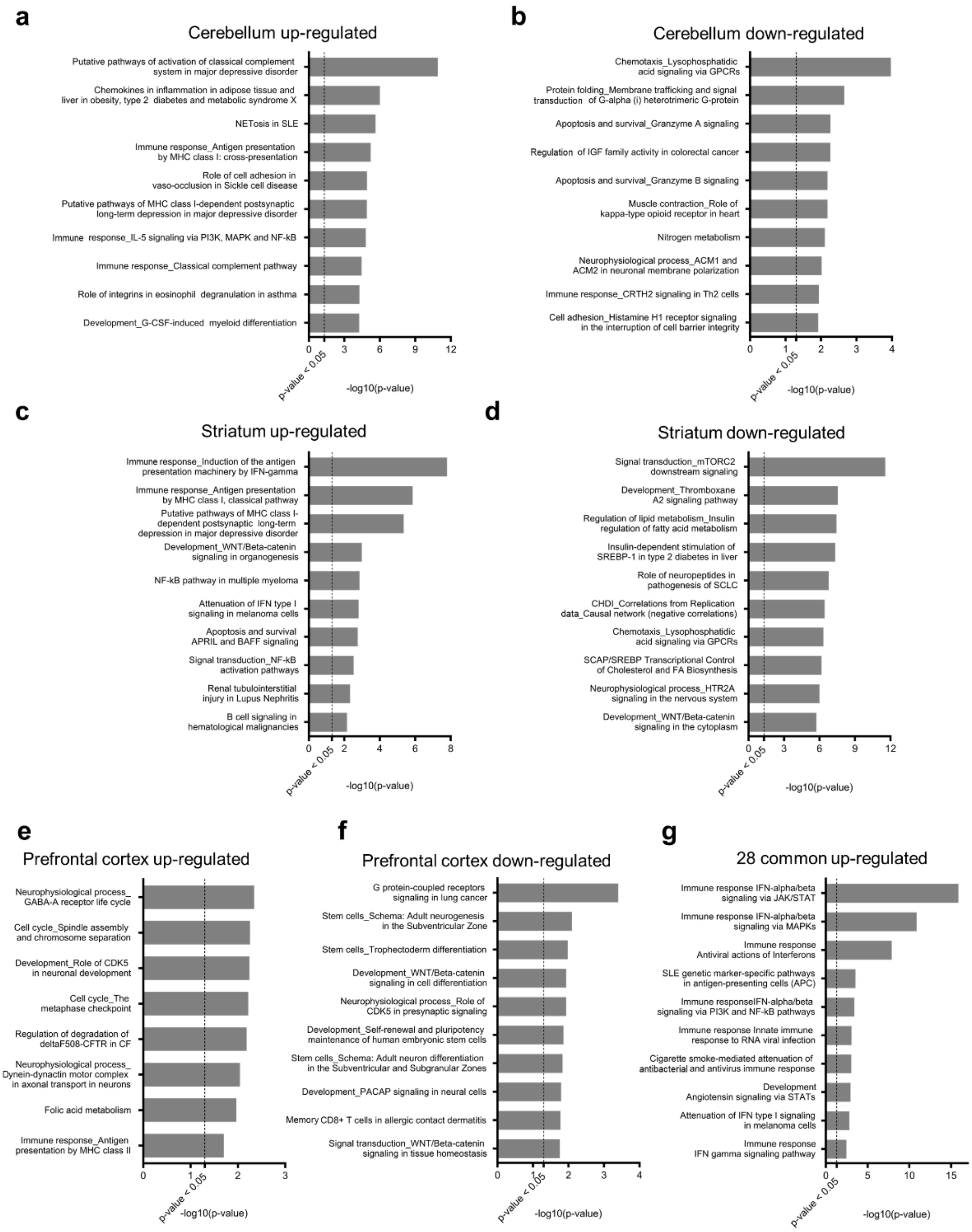

Supplementary figure 3 (continued)

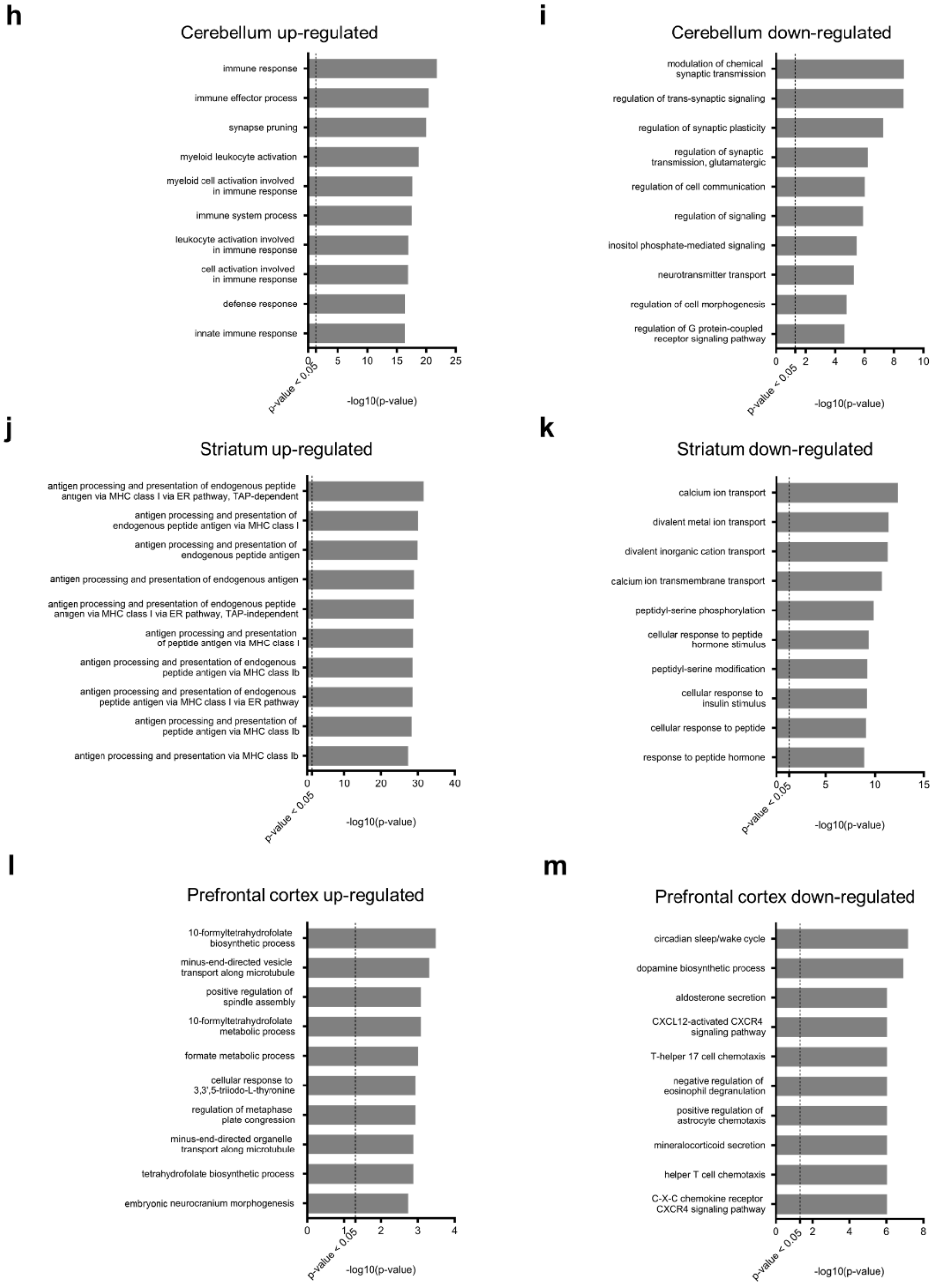

Supplementary Figure 3 Gene enrichment analyses of differentiated expressed genes in the cerebellum, striatum and prefrontal cortex in SCA17 knock-in mice. **a-f** Top 10 enriched pathways of differentiated expressed genes in the cerebellum (a-b), striatum (c-d), and prefrontal cortex (e-f). **g** Common 28 up-regulated genes in three brain regions was enriched in immune related pathways. **h-m** Top 10 enriched GO process of differentiated expressed genes in the cerebellum (h-i), striatum (j-k), and prefrontal cortex (l-m). Source data are provided as a Source Data file.

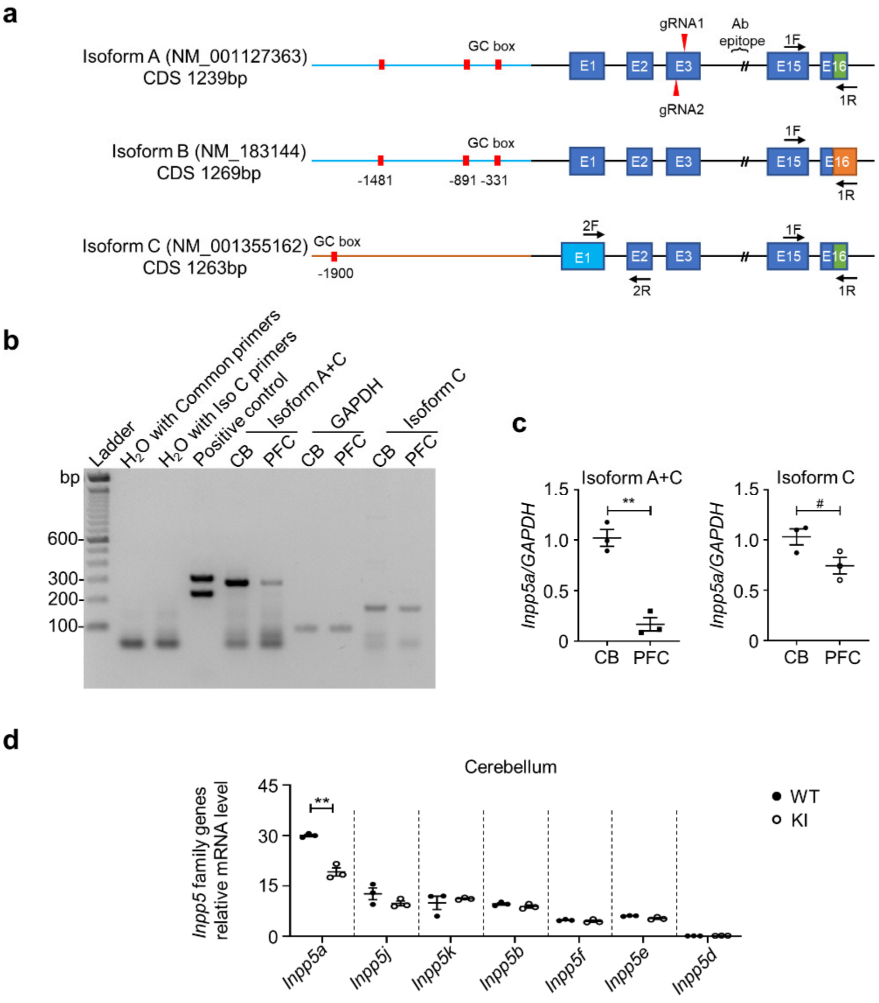

Supplementary Figure 4 Three isoforms of mouse *Inpp5a* gene. **a** Schematic map of three known isoforms of the mouse *Inpp5a* gene. The coding sequences of isoform A, B and C are 1239, 1269 and 1263 bp respectively. Sequence alignment reveals that isoform A and isoform B differ in their C terminus; isoform C has a distinct promoter and exon1 sequence. The location of the GC box (red rectangle), gRNA targeting sites (red triangle), antibody epitope (curly bracket) and PCR primers (arrow) are indicated. **b** Polymerase chain reaction (PCR) analysis using isoform-specific primers showed different expression levels of two *Inpp5a* isoforms in the cerebellum (CB) and prefrontal cortex (PFC). Common primers, Forward 1 (1F): 5'-GCA TCC TCA TGT CCC TGT CT-3' and reverse 1 (1R): 5'-TTA GGA GGA TGA GTT GGA TA-3', amplified 272 bp PCR products for isoform A or C and 221 bp for isoform B. Isoform C specific primers, 2F: 5'- GAA AGA CAT GGC CTG GAG AG-3' and 2R: 5'-GGA GGC CTC GTA GTT TTT CC-3', amplified a 146 bp PCR product. Since there were no 221 bp PCR products, isoform B may not be expressed or may be expressed at an undetectable level in the cerebellum and prefrontal cortex. A positive control (upper band: 300 bp, lower band: 250 bp) demonstrated that the resolution of 2% agarose gel is sufficient to distinguish small bands. **c** Quantification of PCR products. *GAPDH* is used as the internal control. Densitometric ratios of *Inpp5a* to *GAPDH* were normalized to CB, and were analyzed with

Student's t test. t = 2.519, # *P* = 0.0655, t = 8.059, ** *P* = 0.0013, n = 3 mice per group. **d** RNA sequencing identified that *Inpp5a*, but not other *Inpp5* paralogs, was significantly down-regulated in the cerebellum of SCA17 knock-in (KI) mice, compared to wild type (WT) mice. The data were analyzed with Student's t test, t = 8.474, ** *P* = 0.0011. n = 3 mice per group. Data are represented as mean ± SEM. Source data are provided as a Source Data file.

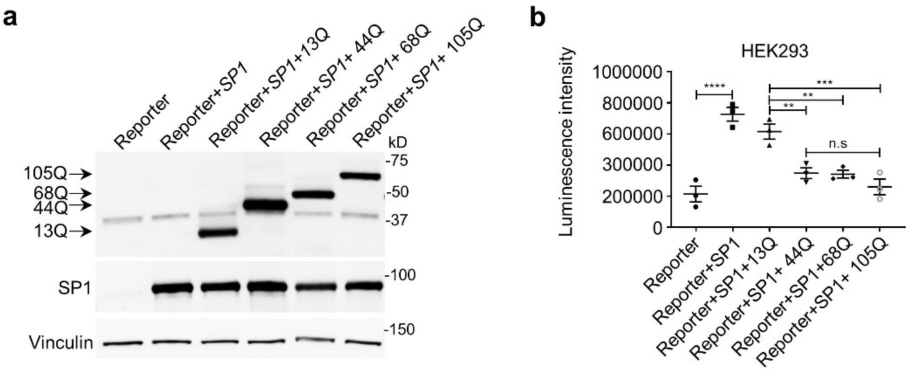

Supplementary Figure 5 Mutant TBP affects SP1 transcriptional activity. **a** Western blotting shows the expression of SP1 with TBP-13Q, -44Q, -68Q, or -105Q in transfected HEK293 cells. **b** Luciferase activity analysis of HEK293 cells transfected with *SP1* and *TBP*-13Q, -44Q, -68Q or -105Q, suggesting that mutant TBP affects SP1's transcriptional activity on *Inpp5a* independent of polyQ repeat length. Luminescence intensity was analyzed with One-way ANNOVA followed with Tukey's multiple comparisons test. F = 22.87, ** $P < 0.005$, *** $P < 0.0005$, **** $P < 0.0001$. Data are represented as mean ± SEM. Source data and full blots are provided as a Source Data file.

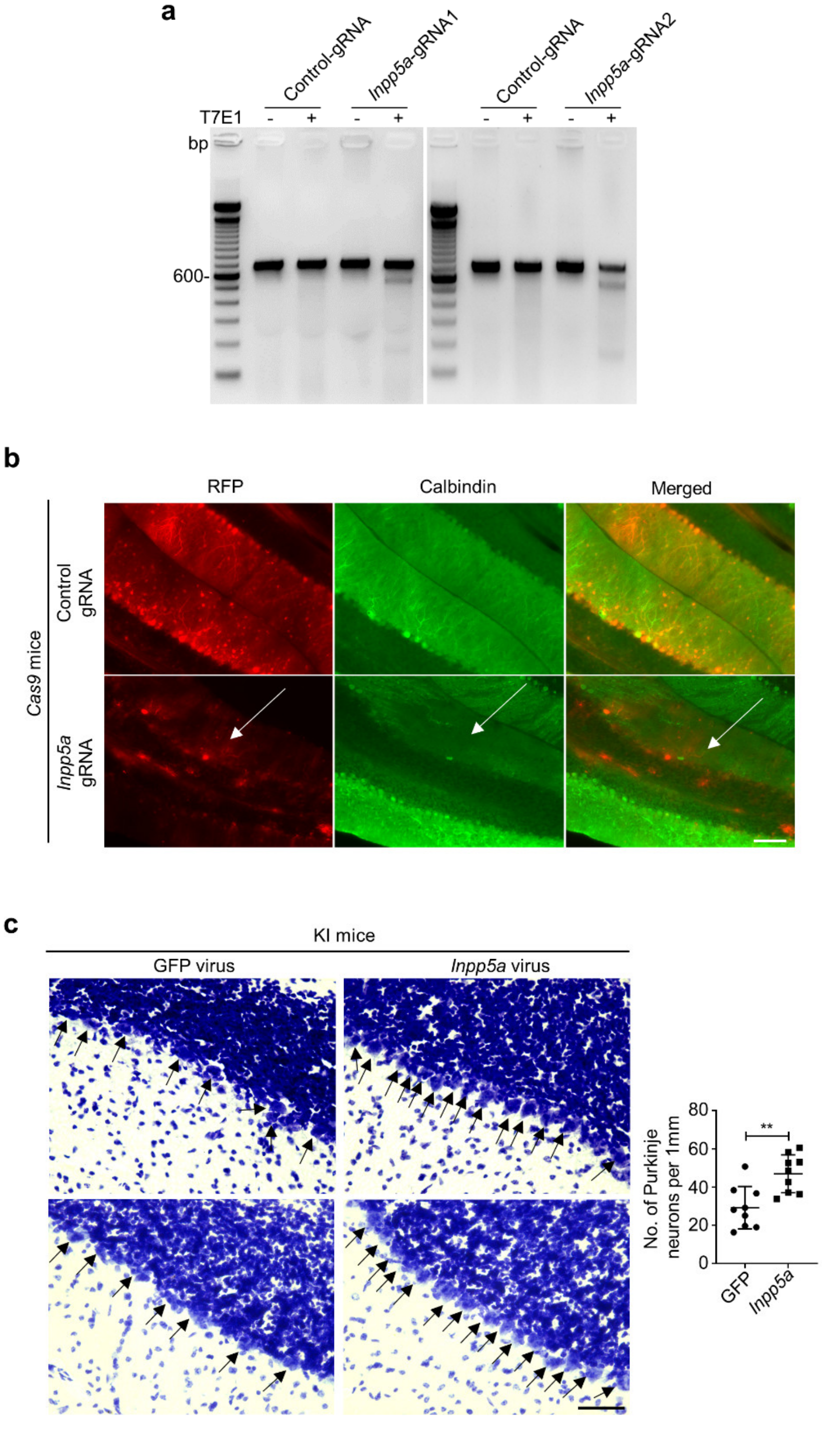

Supplementary Figure 6 Verification of targeting the mouse *Inpp5a* by its gRNA and CRISPR/Cas9. **a** T7 Endonuclease I (T7E1) assay using genomic DNA from Neuro2a cells transfected with *Cas9* plus control gRNA or *Inpp5a* gRNA verified the targeting of the mouse *Inpp5a* gene by its gRNA. **b** Representative immunofluorescence images of AAV-control gRNA- or AAV-*Inpp5a* gRNA-injected cerebellum in *Cas9* transgenic mice without mutant *TBP*. RFP (red) represents viral transduction and gRNA expression. Arrow indicates AAV-*Inpp5a* gRNA-injected region that shows loss of calbindin-labeled Purkinje cells (green) as compared with the region without AAV transduction or with AAV-control gRNA injection.

Scale bar = 100 μm. **c** Representative Nissl staining images (left panel) of AAV-GFP or AAV-*Inpp5a* injected cerebellum in SCA17 mice. Scale bar = 50 μm. Quantification of Purkinje cells (right panel) was analyzed with Student's t test, t = 3.581, ** $P = 0.0025$. Data are represented as mean ± SEM. n = 3 mice per group, three images were used to count from each mouse. Source data are provided as a Source Data file.

Supplementary table 1 Potential GC-Box motifs on the promotor of *Inpp5a* isoform A and B.

Matrix Family	Detailed Family Information	Matrix	Detailed Matrix Information	From	To	Anchor	Strand	Matix sim	Sequence
V$SP1F	GC-Box factors SP1/GC	V$SP 1.01	Stimulating protein 1, ubiquitous zinc finger transcription factor	-899	-883	-891	Forward	0.987	gggagGGGCggggtatg
V$SP1F	GC-Box factors SP1/GC	V$SP 2.01	Sp2, member of the Sp/XKLF transcription factors with three C2H2 zinc fingers in a conserved carboxyl-terminal domain	-32	-16	-24	Forward	0.972	ctgtggggcgGGACttg
V$SP1F	GC-Box factors SP1/GC	V$SP 1.03	Stimulating protein 1, ubiquitous zinc finger transcription factor	-339	-323	-331	Forward	0.958	cggcaGGGCggggcaaa
V$SP1F	GC-Box factors SP1/GC	V$SP 1.03	Stimulating protein 1, ubiquitous zinc finger transcription factor	-1489	-1473	-1481	Forward	0.932	atagcGGGCgggcaaga
