# Supplementary material for: Cerebellum-enriched protein INPP5A contributes to selective neuropathology in mouse model of spinocerebellar ataxias type 17
Source: Nat Commun. 2020 Feb 27;11:1101. doi: 10.1038/s41467-020-14931-8 (PMC7046734; doi:10.1038/s41467-020-14931-8)
Supplement: Supplementary file 3 — Description of Additional Supplementary Information [file 41467_2020_14931_MOESM3_ESM.pdf]

Description of Additional Supplementary Files

File Name: Supplementary Data 1
Description: Differentially expressed genes identified by RNA sequencing in the cerebellum, striatum and prefrontal cortex of SCA17 KI mice
