# Supplementary material for: Cerebellum-enriched protein INPP5A contributes to selective neuropathology in mouse model of spinocerebellar ataxias type 17
Source: Nat Commun. 2020 Feb 27;11:1101. doi: 10.1038/s41467-020-14931-8 (PMC7046734; doi:10.1038/s41467-020-14931-8)
Supplement: Supplementary file 5 — Reporting summary [file 41467_2020_14931_MOESM5_ESM.pdf]

nature research

Corresponding author(s): Shihua Li

Last updated by author(s): Jan 23, 2020

Reporting Summary

Nature Research wishes to improve the reproducibility of the work that we publish. This form provides structure for consistency and transparency in reporting. For further information on Nature Research policies, see Authors & Referees and the Editorial Policy Checklist .

Statistics

For all statistical analyses, confirm that the following items are present in the figure legend, table legend, main text, or Methods section.

n/a	Confirmed	
☐	☒	The exact sample size (*n*) for each experimental group/condition, given as a discrete number and unit of measurement
☐	☒	A statement on whether measurements were taken from distinct samples or whether the same sample was measured repeatedly
☐	☒	The statistical test(s) used AND whether they are one- or two-sided<br>*Only common tests should be described solely by name; describe more complex techniques in the Methods section.*
☒	☐	A description of all covariates tested
☐	☒	A description of any assumptions or corrections, such as tests of normality and adjustment for multiple comparisons
☐	☒	A full description of the statistical parameters including central tendency (e.g. means) or other basic estimates (e.g. regression coefficient) AND variation (e.g. standard deviation) or associated estimates of uncertainty (e.g. confidence intervals)
☐	☒	For null hypothesis testing, the test statistic (e.g. *F*, *t*, *r*) with confidence intervals, effect sizes, degrees of freedom and *P* value noted<br>*Give P values as exact values whenever suitable.*
☒	☐	For Bayesian analysis, information on the choice of priors and Markov chain Monte Carlo settings
☒	☐	For hierarchical and complex designs, identification of the appropriate level for tests and full reporting of outcomes
☒	☐	Estimates of effect sizes (e.g. Cohen's *d*, Pearson's *r*), indicating how they were calculated

Our web collection on statistics for biologists contains articles on many of the points above.

Software and code

Policy information about availability of computer code

Data collection	Softwares used in RNA sequencing were described in methods. Guide RNA was designed by an online tool (https://www.benchling.com/crispr/)
Data analysis	statistical analyses were performed using the Prism 8 (GraphPad) software; Gene enrichment analyses were performed by MetaCore.

For manuscripts utilizing custom algorithms or software that are central to the research but not yet described in published literature, software must be made available to editors/reviewers. We strongly encourage code deposition in a community repository (e.g. GitHub). See the Nature Research guidelines for submitting code & software for further information.

Data

Policy information about availability of data

All manuscripts must include a data availability statement. This statement should provide the following information, where applicable:

- Accession codes, unique identifiers, or web links for publicly available datasets
- A list of figures that have associated raw data
- A description of any restrictions on data availability

We have provided a full data availability statement that meet the requirements.

Field-specific reporting

Please select the one below that is the best fit for your research. If you are not sure, read the appropriate sections before making your selection.

☒ Life sciences ☐ Behavioural & social sciences ☐ Ecological, evolutionary & environmental sciences

For a reference copy of the document with all sections, see nature.com/documents/nr-reporting-summary-flat.pdf

Life sciences study design

All studies must disclose on these points even when the disclosure is negative.

Sample size	Sample size selection was based on published scientific literature so that the size is deemed large enough.
Data exclusions	No data were excluded from the analyses.
Replication	For immunostaining, western blotting, IP3 detection and qPCR, samples from at least three mice were used, and the results were analyzed from at least three independent experiments.
Randomization	Animals used for each experiment are littermates randomly assigned to the experimental groups.
Blinding	RNA sequencing analysis were performed by personnel blinded to the treatment conditions. For western blotting, immunostaining, IP3 detection and qPCR, no blinding were used.

Reporting for specific materials, systems and methods

We require information from authors about some types of materials, experimental systems and methods used in many studies. Here, indicate whether each material, system or method listed is relevant to your study. If you are not sure if a list item applies to your research, read the appropriate section before selecting a response.

Materials & experimental systems

n/a	Involved in the study	
☐	☒	Antibodies
☐	☒	Eukaryotic cell lines
☒	☐	Palaeontology
☐	☒	Animals and other organisms
☒	☐	Human research participants
☒	☐	Clinical data

Methods

n/a	Involved in the study	
☒	☐	ChIP-seq
☒	☐	Flow cytometry
☒	☐	MRI-based neuroimaging

Antibodies

Antibodies used	Primary antibodies used in this study include: 1TBP18 (QED bioscience, 70102), Calbindin (Millipore, ab1778), DARPP32(Abcam, ab40801), NeuN (Cell signaling technology, 24307s), GFP (BD Living Colors, 632376), RFP (Abcam, ab62341), vinculin (Cell signaling technology, 13901), β-actin (Cell signaling technology, 4967L), INPP5A (Invitrogen, PA5-45906), SP1 (Santa Cruz, sc-17824 X). Secondary antibodies were donkey anti-rabbit, donkey anti-mouse Alexa Fluor 488 or 594 from Jackson ImmunoResearch.
Validation	All antibodies used in this study have been validated by manufacturers or validated in published scientific literature.

Eukaryotic cell lines

Policy information about cell lines

Cell line source(s)	Human Embryonic Kidney 293 cells, Neuro2a cells and stable TBP transfected PC12 cell lines.
Authentication	All cell lines were original purchased from ATCC. G418 was added to PC12 cells to screen positive TBP transfected cell lines.
Mycoplasma contamination	All cell lines were tested negative for mycoplasma contamination.
Commonly misidentified lines (See ICLAC register)	No commonly misidentified cell lines were used.

Animals and other organisms

Policy information about studies involving animals; ARRIVE guidelines recommended for reporting animal research

Laboratory animals	Wild type C57BL/6 mice, SCA17 germline knockin mice have been stably maintained in our lab. Cas9 transgenic mice were generated by crossing EIIa-Cre mice with conditional Cas9 mice that were obtained from the Jackson Laboratory . Both male and female mice were involved in this study. The detailed age of mice were described in methods or figure legend.

Wild animals	This study did not involve wild animals
Field-collected samples	All mice were maintained on a 12:12 h light/dark cycle (lights off at 7 p.m.). The temperature was maintained at 22 plus or minus 1 °C with relative humidity (30-70%).
Ethics oversight	All animal procedures were performed in accordance with the NIH and U.S. Public Health Service's Guide for the care and Use of Laboratory Animals, and were approved by the Institutional Animal Care and Use Committee at Emory University, which is accredited by the American Association for Accreditation of Laboratory Care (AAALC).

Note that full information on the approval of the study protocol must also be provided in the manuscript.
